# Group III phospholipase A2 downregulation attenuated survival and metastasis in ovarian cancer and promotes chemo-sensitization

**DOI:** 10.1186/s13046-021-01985-9

**Published:** 2021-06-03

**Authors:** Upasana Ray, Debarshi Roy, Ling Jin, Prabhu Thirusangu, Julie Staub, Yinan Xiao, Eleftheria Kalogera, Andrea E. Wahner Hendrickson, Grace D. Cullen, Krista Goergen, Ann L. Oberg, Viji Shridhar

**Affiliations:** 1 Department of Laboratory Medicine and Pathology, Mayo Clinic, Rochester, MN USA; 2 Alcorn State University, Lorman, MS USA; 3 Division of Gynecologic Surgery, Mayo Clinic, Rochester, MN USA; 4 Department of Internal Medicine, Division of Medical Oncology, Mayo Clinic, Rochester, MN USA; 5 Department of Health Sciences Research, Division of Biomedical Statistics and Informatics, Mayo Clinic, Rochester, MN USA

**Keywords:** Ovarian cancer, metastasis, Group III phospholipase A2, chemosensitivity, autophagy, primary cilia

## Abstract

**Background:**

Aberrant lipogenicity and deregulated autophagy are common in most advanced human cancer and therapeutic strategies to exploit these pathways are currently under consideration. Group III Phospholipase A2 (sPLA2-III/PLA2G3), an atypical secretory PLA2, is recognized as a regulator of lipid metabolism associated with oncogenesis. Though recent studies reveal that high PLA2G3 expression significantly correlates with poor prognosis in several cancers, however, role of PLA2G3 in ovarian cancer (OC) pathogenesis is still undetermined.

**Methods:**

CRISPR-Cas9 and shRNA mediated knockout and knockdown of PLA2G3 in OC cells were used to evaluate lipid droplet (LD) biogenesis by confocal and Transmission electron microscopy analysis, and the cell viability and sensitization of the cells to platinum-mediated cytotoxicity by MTT assay. Regulation of primary ciliation by PLA2G3 downregulation both genetically and by metabolic inhibitor PFK-158 induced autophagy was assessed by immunofluorescence-based confocal analysis and immunoblot. Transient transfection with GFP-RFP-LC3B and confocal analysis was used to assess the autophagic flux in OC cells. PLA2G3 knockout OVCAR5 xenograft in combination with carboplatin on tumor growth and metastasis was assessed *in vivo*. Efficacy of PFK158 alone and with platinum drugs was determined in patient-derived primary ascites cultures expressing PLA2G3 by MTT assay and immunoblot analysis.

**Results:**

Downregulation of PLA2G3 in OVCAR8 and 5 cells inhibited LD biogenesis, decreased growth and sensitized cells to platinum drug mediated cytotoxicity *in vitro* and in *in vivo* OVCAR5 xenograft. PLA2G3 knockdown in HeyA8MDR-resistant cells showed sensitivity to carboplatin treatment. We found that both PFK158 inhibitor-mediated and genetic downregulation of PLA2G3 resulted in increased number of percent ciliated cells and inhibited cancer progression. Mechanistically, we found that PFK158-induced autophagy targeted PLA2G3 to restore primary cilia in OC cells. Of clinical relevance, PFK158 also induces percent ciliated cells in human-derived primary ascites cells and reduces cell viability with sensitization to chemotherapy.

**Conclusions:**

Taken together, our study for the first time emphasizes the role of PLA2G3 in regulating the OC metastasis. This study further suggests the therapeutic potential of targeting phospholipases and/or restoration of PC for future OC treatment and the critical role of PLA2G3 in regulating ciliary function by coordinating interface between lipogenesis and metastasis.

**Supplementary Information:**

The online version contains supplementary material available at 10.1186/s13046-021-01985-9.

## Background

Peritoneal dissemination at diagnosis contribute to poor prognosis in ovarian cancer (OC) [1, 2]. Understanding the molecular alterations that promote the aggressive behavior of OC can lead to new therapeutic options. Transformed cells rewire their metabolism [3] that enables them to survive and adapt to prevailing stress [4]. In addition to an exacerbated glycolysis, transformed cells exhibit adaptations in lipid/cholesterol metabolism to support their high growth rate [5] by scavenging exogenous lipids or by activating endogenous lipogenesis [6, 7]. Excessive lipids/cholesterol stored as lipid droplets (LDs) in malignant cells are considered as one of the hallmarks of tumor aggressiveness conferring chemoresistance [8, 9]. Also, stress-induced FAs released from stored LDs supplied energy to maintain survival and metastatic phenotype of OC cells [10, 11]. Assessment of tumor LD content by Raman spectroscopy is an emerging tool to monitor therapeutic response in patients [12]. Thus adapting novel therapeutic strategies to exploit the lipid-related metabolic dependence in cancer may improve the overall survival.

Phospholipase A_2_ (PLA_2_), a group of enzymes that hydrolyze phospholipids to release fatty acids (FA) and lysophospholipids, are critical regulator of lipid metabolism of transformed cells and associated with cancer progression [13]. Amongst them, Group III sPLA_2_ (PLA2G3) was identified as a candidate biomarker for colon cancer positively correlated with short survival and high lymph node metastasis [14]. The biologically active lipid mediators generated by these enzymes stimulate proliferation abrogate apoptosis, increase inflammation and angiogenesis. Recent studies implicate sPLA_2_s in the regulation of basal lipid metabolism, thus opening a new avenue to uncover the diverse role of this secretory enzyme in cancer [15].

Autophagy maintains the energy balance and cellular homeostasis through turnover of unwanted proteins/organelles in lysosomes [16]. Reports suggest that in addition to acting as substrates for lipases, LDs under stress conditions undergo lipophagy, an autophagy-mediated breakdown [17]. Furthermore, autophagy plays a critical role in regulating primary ciliogenesis depending on cancer types [18, 19]. Primary cilia (PC), a conserved microtubular appendage originating from mother centrosome and extending to extracellular space, are signal hubs in maintaining development and tissue homeostasis [20, 21]. Dysregulated ciliary function is associated with oncogenesis and the loss of ciliogenesis in pre-invasive stages of cancer is considered an early oncogenic event [22–25]. Aberrant activation of lipogenic transcription factor SREBP1c mediates ciliary loss in well-ciliated non-malignant cellular models [26]. However, the connection between lipophagy and ciliogenesis is not well characterized. Our study revealed that PLA2G3 regulates lipogenesis and alters the ciliary process in the OC cells which potentially impacts the oncogenesis. We found that targeting lipogenesis with metabolic inhibitor PFK158 attenuates PLA2G3 expression in an autophagy-dependent manner and restores the PC, thereby counteracting OC progression.

## Materials and methods

### Reagents

PFK158 inhibitor was acquired on MTA from Gossamer Bio (San Diego, CA). Other reagents and antibodies used were listed in Table S1.

### Cell culture

The maintenance and the list of cell lines used in this study are shown in Table S2. Patient-derived ascites were obtained through the Mayo Clinic Ovarian SPORE program (IRB-1288-03, Dr. Shridhar) and in collaboration with the University of Minnesota Cancer Center Tissue Procurement Facility with IRB approval and cultured as mentioned [27, 28].

### Generation of PLA2G3 downregulated stable clones

OVCAR5 stable shRNA (Sigma)-mediated knockdown clones targeting ORF and 3’UTR [sh33:ACCAGTGTGAGCACCAGATTG, sh35:AGTTAGGGATGTCACAGAAAT] and OVCAR8/OVCAR5 stable knockout clone using the sgRNA clone [Target site:GGAGGTCCTGTACCAGCGGA] for hPLA2G3 by CRISPR/Cas9-method (Genecopoeia, USA), were generated following manufacturer protocol.

### Small interfering RNA (siRNA)-mediated transfection

Pooled siRNA against IFT88 and PLA2G3 was used at 20nM concentration as described [29]. Briefly cells seeded in 6-well plates were transiently transfected using the siRNA and lipofectamine reagent for 5-6 h. After 48 h of incubation in media supplemented with 10 %FBS, the cells were harvested for mentioned analysis.

### Cell viability assay

Inhibitory concentrations 50 % (IC50) values were determined using MTT assay in cisplatin (0–40µM), carboplatin (CBP, 0-250µM) treatment and in patient-derived ascites cells upon PFK158-treatment alone or in combination with cisplatin for 24 h. Baflomycin A1 (BafA1,50nM) pretreatment for 2 h followed by dose-dependent cisplatin treatment was performed as mentioned.

### Bodipy staining

Briefly, cells were seeded on an 8-well chambered slide and treated as mentioned for 24 h, washed, and fixed in 4 % paraformaldehyde at room temperature for 10 min followed by 1 µg/ml BODIPY staining for 10 min. Slides were washed with PBS and mounted using Prolong Gold Antifade Reagent with DAPI and examined under Zeiss-LSM 510 fluorescence microscope [30].

### Transmission electron microscopy (TEM) analysis

SCG control-transfected and PLA2G3-KO OVCAR8 cells were fixed in Trump’s fixative [31] and directed to Microscopy and Cell Analysis Core facility in Mayo Clinic, MN for further processing. Images were captured in JEM-1400 TEM (Jeol, USA).

### Immunoblot assay

Cell lysates were subjected to immunoblot [29] with primary antibodies listed in Table S1. Target proteins were visualized by fluorophore-conjugated secondary antibodies (LICOR) and using LI-COR OdysseyFc Imaging System (Nebraska, USA). PCNA was used as loading control in most of the western blot assay as it is a highly conserved protein involved in DNA replication and does not vary with metabolic or cell cycle status and all the western blots performed here were with the whole cell lysates.

### Immunofluorescence (IF) assay

PFK158-treated cells with/without BafA1 or the PLA2G3 KD cells were grown in 8-well chambered slide, fixed and stained with tagged anti-acetylated α-tubulin-Alexa-Fluor561 and anti-PLA2G3 (1:100) as described [31] and visualized by Zeiss-LSM510 confocal microscope. Autophagic flux was measured by confocal microscopy upon transient transfection with RFP-LAMP2 and GFP-LC3B in the KO and SCG-control cells by cisplatin treatment and with GFP-RFP-LC3B followed by PFK158-treatment.

### Cyto-ID autophagy detection by fluorescence microscopy

PLA2G3 KO and SCG-control cells were treated with Cisplatin and assessed for autophagic induction by Cyto-ID staining as per manufacturer’s protocol.

### Immunohistochemistry

The tissues were fixed in formalin, paraffin-embedded and microtome-sectioned. For the IHC staining studies, the tissue sections were deparaffinized and processed followed by probing against Ki-67 as described [32].

### Scratch wound-healing assay

PLA2G3 KD/KO, IFT88KD and respective control-transfected cells were scratched using a 10µL pipette tip and replaced with fresh medium [33]. After 24/48hrs images were captured using EVOS inverted microscope (ThermoFischer Scientific) and represented.

### Clonogenic assay

PLA2G3 KD/KO and control-transfected cells were plated (500cells/well) and allowed to grow for ~14days until colonies became visible. Cells fixed, stained with crystal violet and images provided.

### *In vivo* xenograft study

Female athymic nude mice (nu/nu, 4–6weeks old; Jackson Laboratories, USA) were randomized into 4 groups (*n* = 7). OVCAR5 SCG control-transfected and PLA2G3 KO cells (5 × 10^6^ cells; 2 groups each) were injected intraperitoneally (i.p). Seven days following injection, one group each from the SCG and KO cohorts was treated with CBP (50 mg/kg) once in a week for 4 consecutive weeks. All mice euthanized on day30 and tumor weight was determined. Tumors were preserved in formalin for IHC and fresh frozen for protein analysis. The experiments were carried out under the approved protocol and guidelines of Mayo Clinic Animal Care and Use Committee, MN.

### Statistical analysis

All investigation was performed in triplicates for 3 independent experiments unless mentioned. The results were expressed as mean ± standard deviation. Significant changes (*p < 0.05, **p < 0.01) were determined using student’s t-test unless otherwise noted.

## Results

### PLA2G3-deficient OC cells showed attenuated tumor progression

**Fig. 1:**
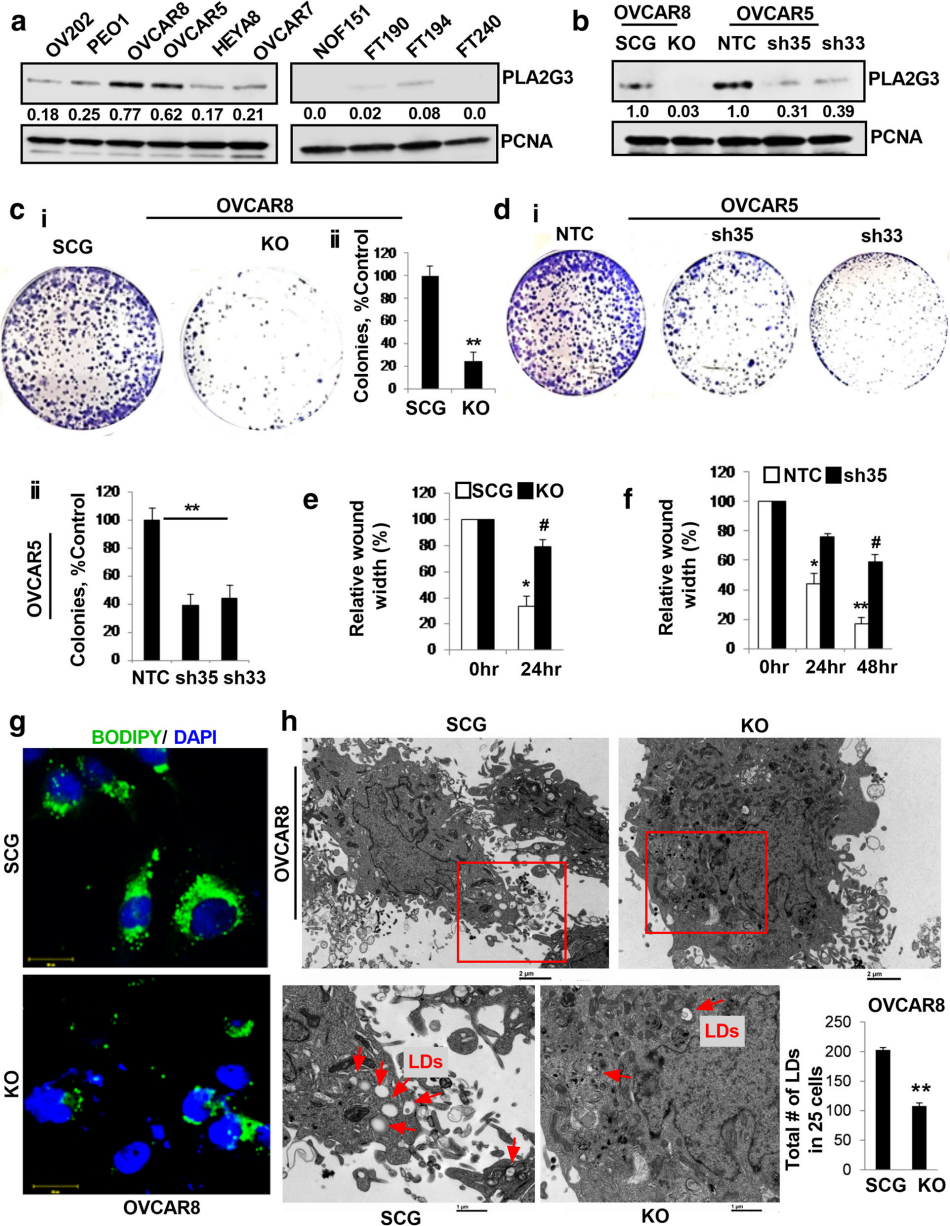
PLA2G3 is associated with increased proliferation, migration and lipogenesis in OC cells. (A) Expression analysis of PLA2G3 by western blot in OC cell lines and the normal hTERT immortalized ovarian fibroblast cell line NOF15hTERT, fallopian tube epithelial cell lines FT 190, 194 and 240. (B) Western blot analysis of PLA2G3 in the SCG and PLA2G3 CRISPR knock out (KO) OVCAR8 cells and in OVCAR5 NTC, sh33 and sh35 PLA2G3 KD clones with PCNA as a loading control. Densitometric analysis showing fold change was calculated using Image J software, normalized to PCNA is provided beneath the panel. (C) Colony forming abilities was assessed in OVCAR8 KO and SCG-control cells and (D) in OVCAR5 NTC control, sh35 and sh33 KD cells. The number of colonies was counted and plotted as mean ± SD (*n* = 3, C-D ii, **p < 0.01 vs. control). Wound healing assay was performed in (E) OVCAR8 KO and (F) OVCAR5 sh35 and sh33 KD cells along with their respective controls and the relative wound width was calculated by Image J software and plotted (*p < 0.05,**p < 0.01 vs. 0 h time point and #*p* < 0.05 control vs. KO/KD cells at 24 and/or 48 h). (G) Representative confocal images of Bodipy stained LDs in OVCAR8 SCG and PLA2G3 KO cells. (H) Representative TEM images of OVCAR8 SCG and KO cells showing the LDs (red arrows) were provided. Scale bar: 2 and 1 μm respectively. Quantification of the LDs per 25 cells was plotted, **p < 0.01

Immunoblot analysis showed PLA2G3 is highly expressed in several OC cells compared to normal fallopian tube epithelial cells FTs 190 and 194 and is not expressed either in FT240 [34] or normal ovarian fibroblast NOF151hTERT cells (Fig. 1 A). TCGA analysis of high-grade serous subtype showed 2.26 % cases with altered PLA2G3 expression (@cbioportal, Fig. S1A). To better understand the role of PLA2G3, we generated PLA2G3 knockout (KO) clone in OVCAR8 cells and two shRNA-mediated stable PLA2G3-KD clones of OVCAR5 (sh33/sh35) along with scrambled RNA (SCG) and non-targeted control (NTC) transduced cells as controls respectively. Efficient PLA2G3 downregulation in the KO and KD cells was verified by immunoblot analysis (Fig. 1B).

Clonogenic survival assays of OVCAR8-KO and OVCAR5-sh33/sh35 KD cells showed significantly reduced number of colonies compared to respective controls (Fig. 1 C-D). Additionally, wound-healing assay showed significant reduced migration in both KO and KD cells compared to respective controls (Fig. 1E-F, S1B-C). We reported that group-IVA cytosolic phospholipase A2 is a critical regulator for LD biogenesis in cancer [35]. To determine whether PLA2G3 affects lipid metabolism in OC cells, we assessed LD formation by Bodipy staining and found a decrease in the number of LDs in KO and sh35/33 KD cells compared to respective controls (Fig. 1G, S1D). Consistent with these results, TEM analysis also showed a significant reduction in the numbers of LDs in the OVCAR8-KO cells compared to SCG-transfected cells (Fig. 1 H). Collectively, these results suggest that PLA2G3 abrogation impairs the lipogenesis pathway and attenuates OC tumorigenesis.

### PLA2G3 KD cells are sensitive to Platinum based-drug treatment

**Fig. 2:**
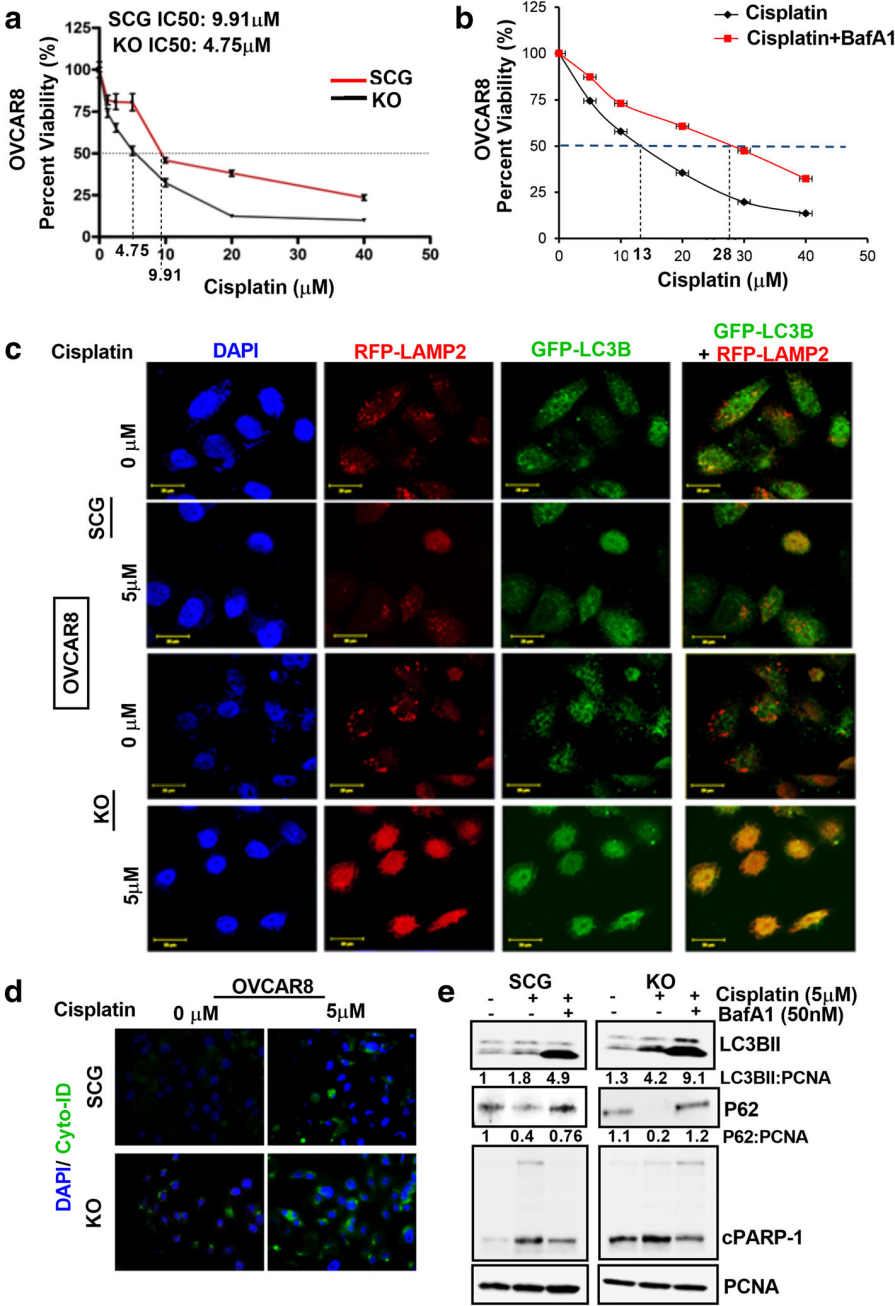
Downregulation of PLA2G3 sensitizes cells to cisplatin-mediated cytotoxicity. (A) Percent cell viability was assessed by MTT assay with an increasing concentration of cisplatin (0–40µM) in OVCAR8 SCG and PLA2G3 KO cells. IC50 value in both the treatment sets was calculated and represented. (B) MTT assay was performed to calculate the percent cell viability with an increasing concentration of cisplatin (0–40µM) for 24 h with and without pretreatment with BafA1 (50nM) for 2 h in OVCAR8 cells and represented. (C) Representative confocal images of 5µM cisplatin (24 h) induced autophagy in OVCAR8 PLA2G3 KO cells compared to SCG control cells upon transient transfection with GFP-LC3B and RFP-LAMP2. Merged images indicate autophagy induction in the KO cells. Scale bar: 20 μm. (D) Cyto-ID staining in the OVCAR8 SCG and PLA2G3 KO cells show the induction of autophagy upon treatment with 5 µM cisplatin for 24 h. DAPI stained nuclei are in blue. (E) Western blot analysis of LC3BII, p62/SQSTM1 and cleaved PARP1 following 5µM cisplatin treatment for 24 h with or without 2 h pretreatment with BafA1 (50nM). PCNA was probed for endogenous control

Given that LD-rich cancer cells exhibit chemo-resistive properties, we investigated whether PLA2G3 KD sensitizes cancer cells to cisplatin/carboplatin-induced cytotoxicity. The OVCAR8 KO and SCG-control cells treated with increasing concentrations of cisplatin for 24 h showed a significant reduction in IC50 value from 9.91µM in SCG-transfected cells to 4.75µM in the KO cells (Fig. 2 A). Similarly, stable HeyA8MDR PLA2G3-KD cells (Fig. S2A) treated with increasing CBP doses for 24 h showed a decrease in IC50 value to 95.4µM compared to 170µM in the NTC cells (Fig. S2B-C). Additionally, the KD cells showed an improved dose-dependent decrease in cell survival upon CBP treatment compared to NTC cells (Fig. S2D). Together, these results substantiate that PLA2G3 downregulation sensitizes cells to platinum-drug mediated cell death.

Likewise, to understand whether induction of autophagy is essential for sensitizing OC cells to cisplatin-induced cytotoxicity, we pretreated OVCAR8 cells with BafA1 (inhibitor of autophagolysosome formation) for 2 h followed by cisplatin treatment for 24 h. Results showed that inhibition of autophagy diminished the cytotoxic effect of cisplatin in OC cells (IC50: 13µM to 28µM, Fig. 2B), which suggests that autophagy-mediated cytotoxicity is critical for sensitizing cancer cells to the platinum drug-induced cell death. The differences in cisplatin sensitivity between parental and SCG transfected OVCAR8 cells could be attributed to the generation of stable clones with antibiotic selection process in the SCG cells, making them vulnerable to the treatment. To validate the role of PLA2G3 in sensitizing cells to platinum drug-induced cytotoxicity, IF analysis for GFP-LC3B puncta expression and its co-localization with lysosomal associated membrane protein 2 (RFP-LAMP2) upon cisplatin treatment in both KO and SCG-transfected OVCAR8 cells was performed. Results showed increased expression and co-localization of RFP-LAMP2 and GFP-LC3B in the KO cells compared to control upon cisplatin treatment (Fig. 2 C). Likewise, Cyto-ID staining used as a read-out for autophagic induction, showed a significant increase in fluorescent signal in KO cells compared to SCG-transfected cells upon treatment with 5µM cisplatin (Fig. 2D). Immunoblot analysis also revealed an increased expression of LC3BII and cleaved PARP1 with reduced p62/SQSTM1 levels in KO cells compared to SCG-transfected cells upon cisplatin treatment (Fig. 2E). Pretreatment for 2 h with BafA1 inhibited the cisplatin-induced autophagy with increase of the LC3BII and rescue of the p62/SQSTM1 levels respectively and a decreased induction of the cleaved PARP1 in the cells (Fig. 2E). Collectively, these results suggest that PLA2G3 KD increased sensitivity of the cancer cells to autophagy-induced cytotoxicity upon treatment with platinum drugs.

### Aberrant PLA2G3 expression impairs PC formation in OC cells

**Fig. 3:**
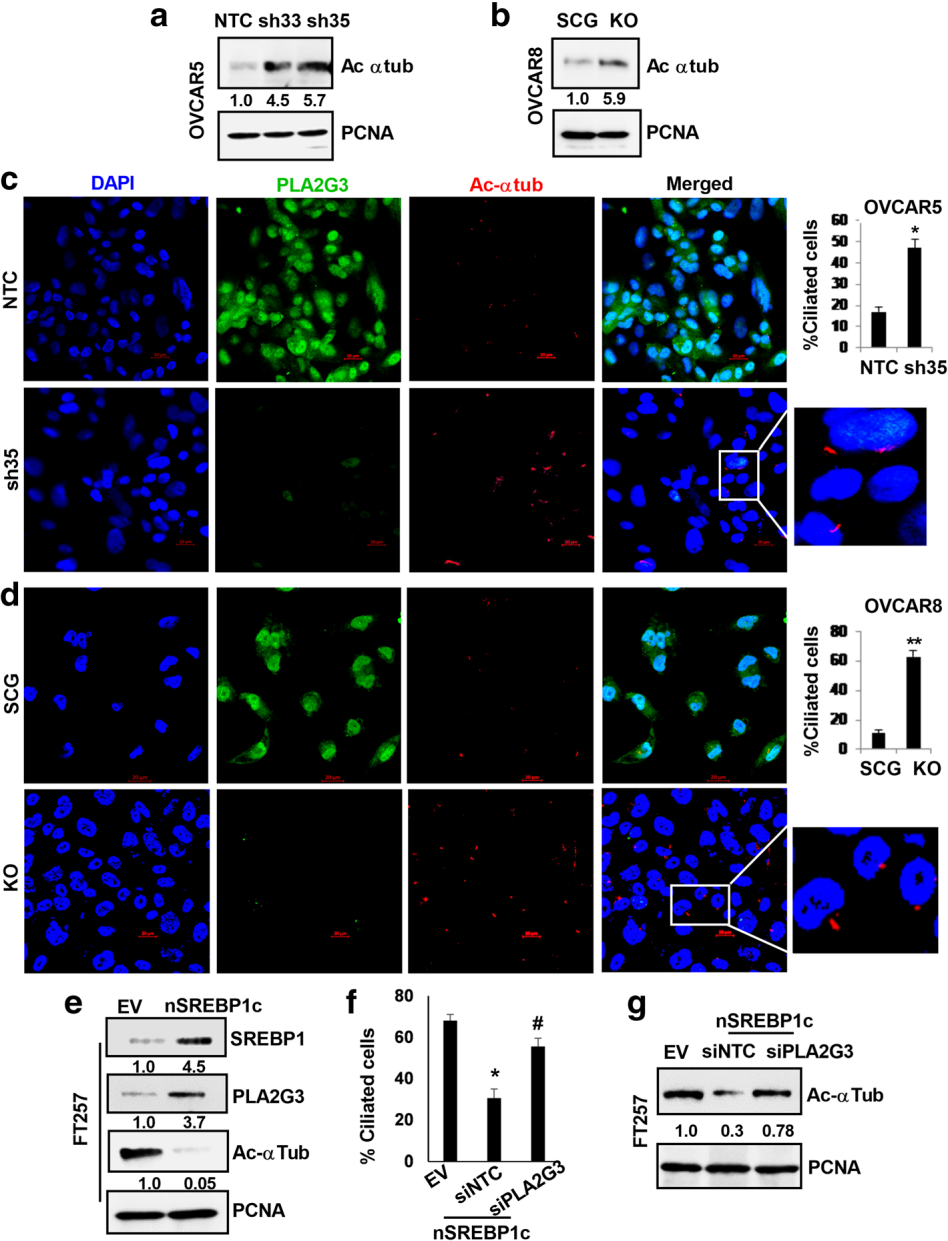
Downregulation of PLA2G3 expression impairs PC formation in OC cells. Western blot analysis of acetylated α tubulin levels in (A) OVCAR5 NTC and sh35, sh33 KD cells and in (B) OVCAR8 SCG and KO cells was shown with PCNA as a loading control. Densitometric analysis indicates the fold change calculated using Image J software, normalized to PCNA and provided beneath the panel. (C) Primary cilia (PC) were detected by IF using fluorescently tagged-acetylated α-tubulin (red) in OVCAR5 NTC and sh35 KD cells. PLA2G3 expression (green) was also assessed in the same cells. Nuclei were stained with DAPI. Scale bar: 20 μm. Quantification, 100 cells per field were scored and the percent ciliated cell was plotted as mean ± SD and shown next to the IF images (*p < 0.05 vs. control). (D) Similar IF study was performed in the OVCAR8 SCG and PLA2G3 KO cells as represented, and percent ciliated cell was plotted (**p < 0.01 vs. control). Scale bar: 20 μm. (E) Immunoblot analysis of SREBP1c, PLA2G3 and acetylated α tubulin levels in the SREBP1c overexpressed FT257 cells with PCNA as a loading control was shown. Densitometric analysis using Image J software was calculated, normalized to PCNA, and the fold change was provided beneath the panel. (G) PC was detected by IF using fluorescently tagged-acetylated α-tubulin (red) in empty vector (EV)-transfected control, nSREBP1c overexpressed FT257 cells in combination with nontargeting siRNA (siNTC) and the siPLA2G3 and the percent ciliated cell was plotted as mean ± SD (EV vs. SREBP1c overexpressed *p < 0.05; siNTC vs. siPLA2G3 in the SREBP1c overexpressed cells #*p* < 0.05). (G) Immunoblot analysis for acetylated α tubulin was also performed in the similar treated cells and shown with PCNA as a loading control. Fold change calculated using Image J software was provided beneath the panel

Since aberrant lipogenic signaling is associated with distortion of PC [26] we assessed whether LD deregulation due to aberrant PLA2G3 expression is involved in regulation of ciliogenesis in OC cells. Immunoblot analysis of OVCAR5-KD and OVCAR8-KO cells showed a significant increase in expression of acetylated α-tubulin (a marker for PC) compared to respective controls (Fig. 3 A-B). Likewise, IF study using fluorescently tagged-acetylated α-tubulin revealed an increase in percent ciliation in OVCAR5 sh35-KD and OVCAR8-KO cells compared to controls (Fig. 3 C-D respectively) with efficient downregulation of PLA2G3 under similar conditions. Together these data validate the importance of PLA2G3 in the regulation of lipid metabolic pathway and PC in OC. To determine the role of PC in OC progression, we transiently knockdown IFT88, a key factor regulating ciliogenesis, and as shown in fig. S3A, a decrease in acetylated α-tubulin in KD cells was confirmed by immunoblot. IFT88 KD showed a significant increase in colony forming ability (Fig. S3B-C) and increased migration of the OVCAR5 cells (Fig. S3D-E).

Further to get an understanding on the consequence of PLA2G3 upregulation, we transiently overexpressed SREBP1c [26] in the FT257 fallopian tube epithelial cells having very low expression of both SREBP1c and PLA2G3 (Fig. S3F-G). Immunoblot analysis showed significant increase in PLA2G3 expression with decreased acetylated α-tubulin levels in the SREBP1c overexpressed cells compared to empty vector (EV) transfected controls (Fig. 3E). IF study using fluorescently tagged-acetylated α-tubulin revealed SREBP1c overexpressed FT257 cells showed reduced PC compared to control (Fig. 3 F and S3H). Additionally, when we transiently KD PLA2G3 by using specific siRNA in the SREBP1c overexpressed cells, there is an increase in percent ciliation compared to the siNTC control (Fig. 3 F and S3H). Similar observation was also confirmed in the western blot analysis of the mentioned cells (Fig. 3G).

### Knockdown of PLA2G3 inhibits ***in vivo*** tumor progression and metastatic spread in OVCAR5 xenograft model

**Fig. 4:**
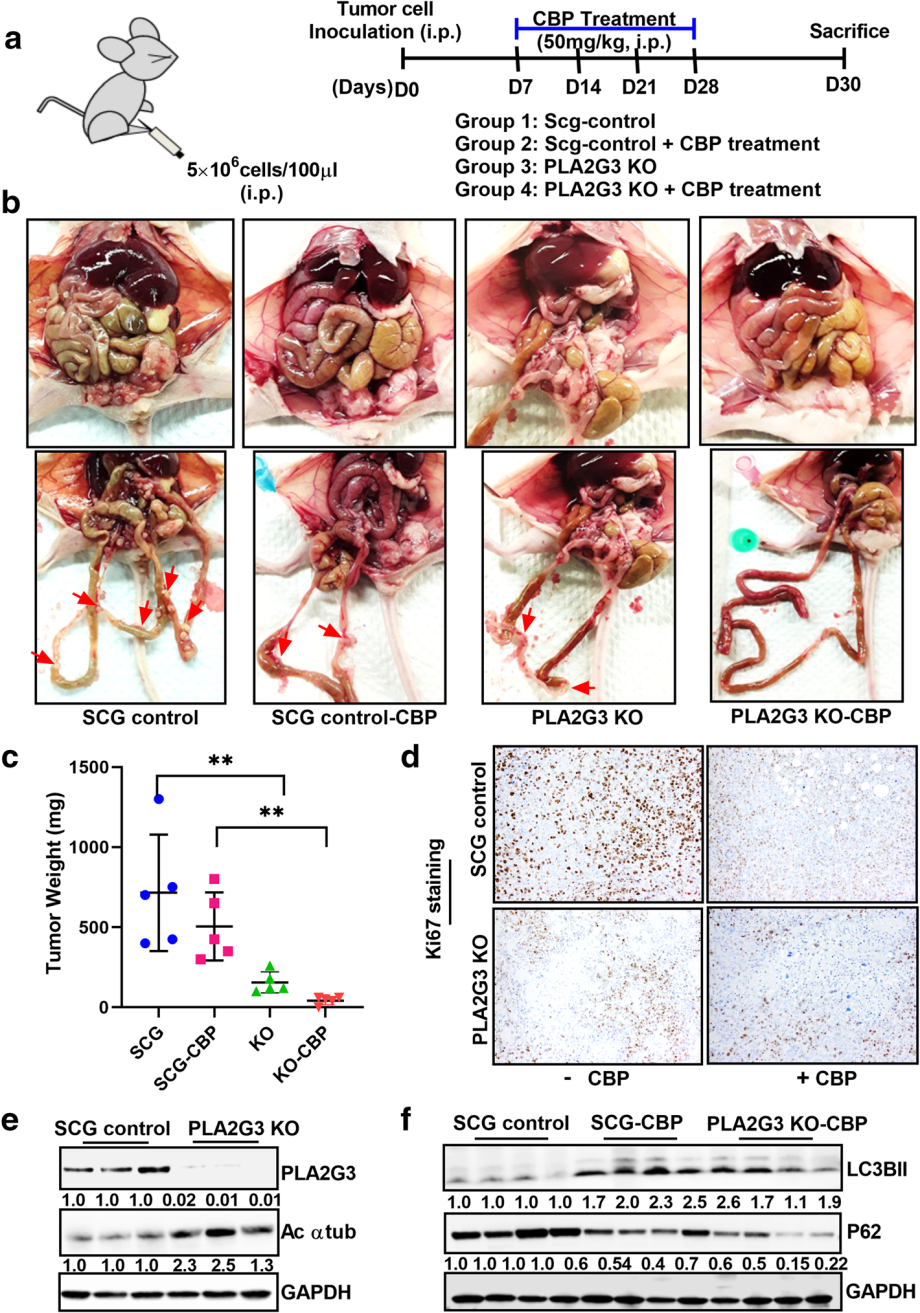
PLA2G3 KD cells are sensitized to carboplatin treatment ***in vivo *in OC xenograft.** (A) Schematic representation of the study model in mice OVCAR5 OC xenograft was provided. (B) Randomized OVCAR5 SCG-transfected control and PLA2G3 KO tumor-bearing mice were treated with or without CBP (50 mg/kg) for 4 weeks at the interval of 7 days. All mice were sacrificed on day 30. Illustrative images of the mice with the tumor burden and metastatic nodes were shown. Arrows point to the metastatic tumor nodules in the differently treated cohort of the animals. (C) Graphical presentation of the excised tumor weights in the treatment cohorts (**p < 0.01). (D) Representative images of IHC staining of Ki67 in the tissue blocks of the four treatment groups. (E) Immunoblot analysis of PLA2G3 and acetylated α-tubulin expression from the lysates of the SCG-control and PLA2G3-KO treated groups. (F) Western analysis of LC3BII and p62/SQSTM1 from the lysates of the SCG-control and CBP treated groups were shown. GAPDH is used as a loading control. Densitometric analysis using Image J software was calculated, normalized to GAPDH, and fold change was provided beneath the panel in both cases

To support our *in vitro* findings, the effect of PLA2G3-KO alone and in combination with CBP treatment on tumor growth and metastatic spread was assessed *in vivo* as described (Fig. 4 A). No significant alteration in health condition was observed (data not shown); however, two mice died in the control group due to unknown reasons very early on and therefore had to be excluded in the analysis. PLA2G3-KO tumor-bearing mice showed a significant reduction in tumor growth and metastatic spread compared to the SCG-control group (Fig. 4B). Interestingly, the KO tumor-bearing mice showed almost no tumor burden upon CBP treatment compared to the SCG-control cohort (Fig. 4B). Comparative statistical analysis of the tumor weight and Ki67 staining of tumor tissue sections showed a similar significant reduction in the KO model both with and without CBP treatment (Fig. 4 C-D). Immunoblot analysis confirmed downregulated PLA2G3 expression and increased acetylated α-tubulin in the KO-cohort compared to SCG-derived xenografts (Fig. 4E) and an increased expression of LC3BII with p62 downregulation in CBP treated SCG-control and KO cohort of mice (Fig. 4 F). Hence, our *in vivo* data supports the role of PLA2G3 in metastatic spread and its downregulation sensitizes cells to chemotherapy.

### Targeting by PFK158 inhibitor restores PC by reducing PLA2G3 in an autophagy-dependent manner

**Fig. 5:**
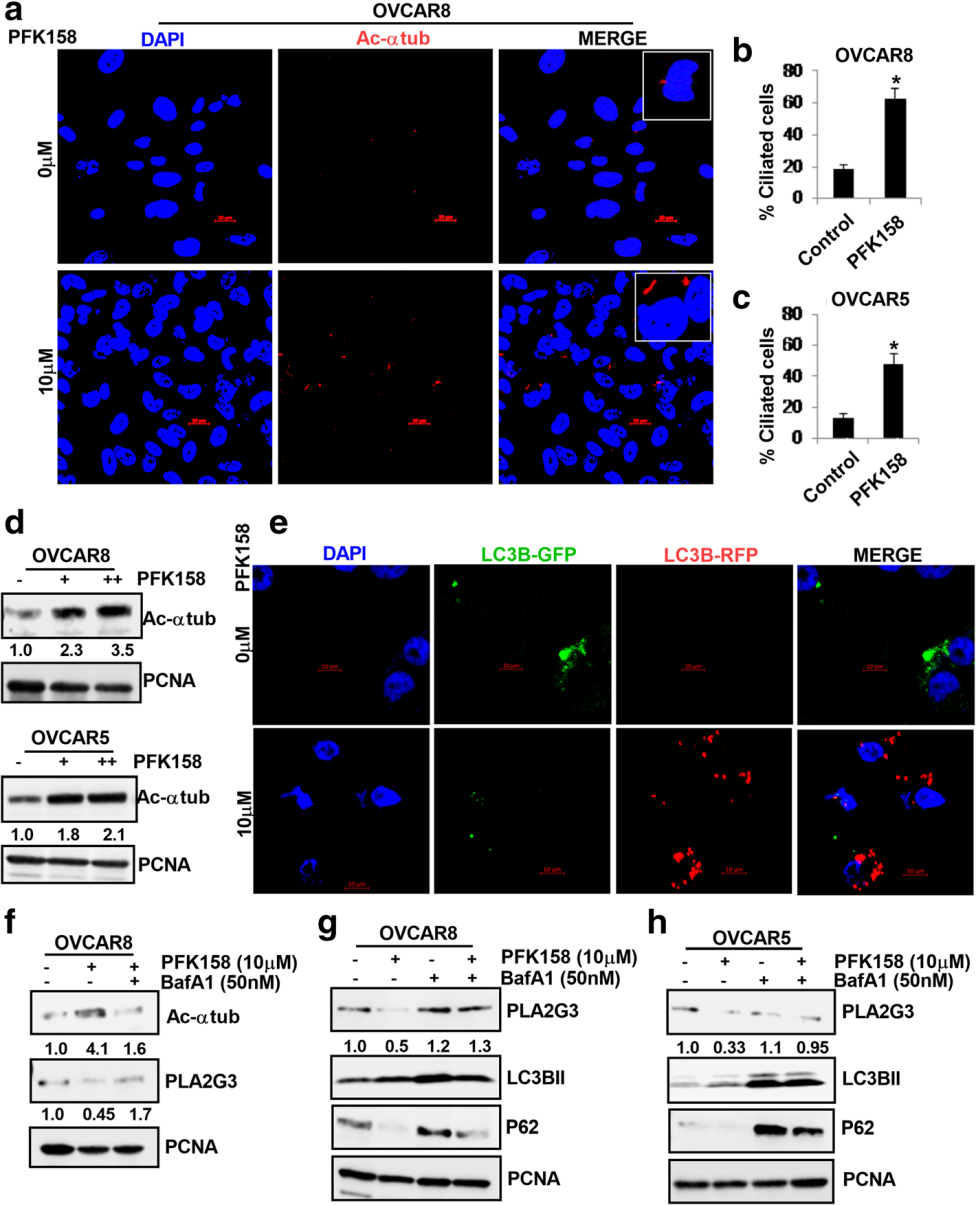
PFK158 induces PC by targeting PLA2G3 in an autophagy-dependent manner in OC cells. (A) Representative confocal IF images of fluorescently tagged-acetylated α tubulin (red) indicate percent ciliated cells upon treatment with 10µM PFK158 for 24 h in OVCAR8 cells. Nuclei were stained with DAPI. Scale Bar: 20 μm. (B) In OVCAR8 and (C) OVCAR5 the percent ciliated cells were quantified in 100 cells per field from the IF study and represented as mean ± SD (*p < 0.05 vs. control). (D) Western blot analysis shows the levels of acetylated α-tubulin in both OVCAR8 and 5 cells upon treatment with 5 and 10 µM PFK158 for 24 h. Fold change was calculated using the Image J software, normalized to PCNA endogenous control and provided beneath the panel. (E) IF study of autophagic flux induction (GFP + to RFP + GFP-) in 10µM PFK158 treated OVCAR5 cells for 24 h was performed after transient expression of GFP-RFP-LC3B plasmid. Scale Bar: 10 μm. (F) Immunoblot analysis was performed to show the levels of acetylated α-tubulin and PLA2G3 in the OVCAR8 cells upon treatment with 10µM PFK158 with or without 50nM BafA1 pretreatment (2 h). (G) PLA2G3, LC3BII and p62/SQSTM1 levels were analyzed by western blot in the 10µM PFK158-treated OVCAR8 cells with or without 2 h pretreatment with BafA1 (50nM). (H) Similar analysis was performed in the OVCAR5 cells. PCNA was used as a loading control. Fold change was calculated using the Image J software, normalized to PCNA endogenous control and provided beneath the panel

Although we highlighted the role of PFK158-induced autophagy in regulating lipophagy [35], it did not address if PFK158 regulated ciliation. Driven by our observations, we wondered if inhibition of lipogenic signaling by PFK158 can restore ciliation in OC cells. IF analysis with fluorescently tagged-acetylated α-tubulin, showed that PFK158 treatment significantly restored PC in both OVCAR8 and OVCAR5 cells (Fig. 5 A, S4A). Quantitation of increase in percent cilia is shown in Fig. 5B-C. Immunoblot analysis of acetylated α-tubulin also showed increased expression in OVCAR8 and OVCAR5 cells upon treatment with PFK158 (Fig. 5D). To understand if PFK158-induced autophagy plays a role in induction of PC, we monitored induction of autophagic flux by GFP-RFP-LC3B transfection in PFK158 treated OVCAR5 cells. Confocal analysis showed induction of autophagic flux through the formation of increased red puncta in PFK158-treated cells compared to untreated cells (Fig. 5E). Interestingly, pretreatment with BafA1 for 2 h inhibited PFK158-induced increase of acetylated α-tubulin in OVCAR8 cells (Fig. 5 F, top-panel). Also, immunoblot analysis showed PFK158-treatment attenuated PLA2G3 expression which was restored when cells were pretreated with BafA1 (Fig. 5 F, middle-panel). To understand whether inhibition of autophagy regulates PC levels, we treated OVCAR8 cells with 3MA and BafA1 individually and observed that both early and late stage inhibition of autophagy downregulated acetylated α-tubulin levels (Fig. S4B). Effect of BafA1 as an inhibitor of PFK158-induced autophagy was confirmed by the resulting increases in LC3BII expression and the rescue of p62/SQSTM1 in both the OC cells (Fig. 5G-H; panels2-3). Under similar conditions, the PFK158-induced reduction of PLA2G3 was restored in both cells in presence of BafA1 (Fig. 5G-H; panel-1).

**Fig. 6:**
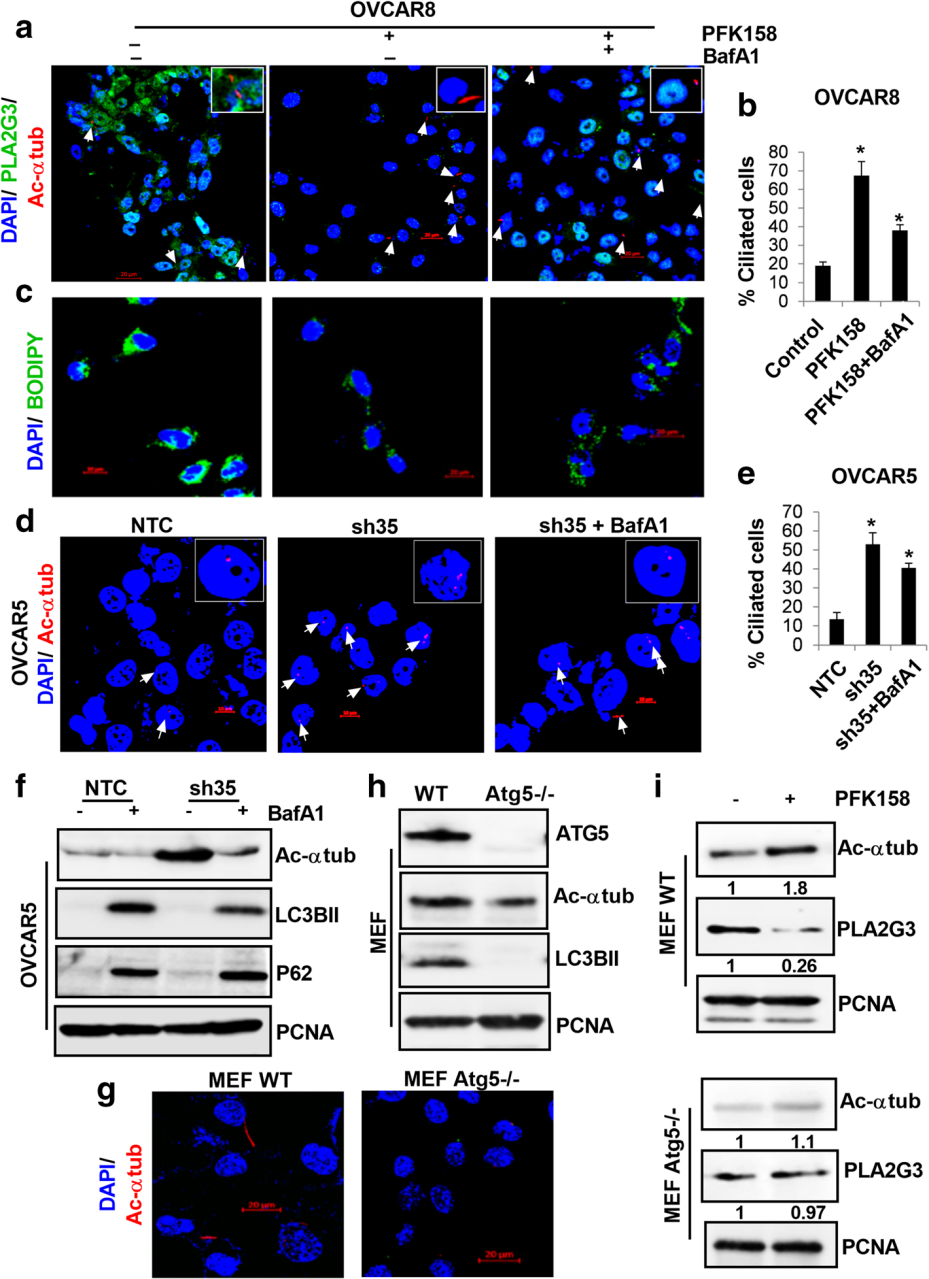
Autophagy plays a critical role in the regulation of PLA2G3 and primary ciliation. (A) Representative confocal images by IF analysis of fluorescently tagged-acetylated α tubulin (red) and PLA2G3 expression (green) upon 10µM PFK158 treatment with or without BafA1 (50nM) pretreatment in OVCAR8 cells. DAPI was used to stain nuclei. Scale bar: 20 μm. (B) Percent ciliated cells was quantified in 100 cells per field and represented as mean ± SD (*p < 0.05 vs. control). (C) Under similar conditions, Bodipy staining of LDs were visualized in OVCAR8 cells. DAPI was used to stain nuclei. Scale bar:20 μm. (D-E) IF study was performed in OVCAR5 NTC and sh35 KD cells in with or without 50nM BafA1 pretreatment (2 h) against fluorescently tagged-acetylated α tubulin (red). DAPI was used to stain nuclei. Scale bar: 10 μm. Percent ciliated cells were scored in 100 cells per field and represented (*p < 0.05 vs. control). (F) Immunoblot analysis of acetylated α tubulin, LC3BII and p62/SQSTM1 under similar condition was performed. PCNA was probed for endogenous control. (G) IF study of acetylated α tubulin levels was assessed in wild-type (WT) and Atg5 knockout MEF cells. Scale bar:20 μm. (H) Western analysis for acetylated α tubulin and LC3BII was done in the mentioned cells. The ATG5 expression level was validated. (I) PLA2G3 and acetylated α tubulin expression was assessed in WT and Atg5-/- MEF cells upon 5µM PFK158 treatment. PCNA was used as a loading control. Fold change was calculated using Image J software, normalized to control and provided beneath the panel

Consistent with these results, PFK158-induced ciliation was inhibited by BafA1 treatment as determined by levels of acetylated α-tubulin with confocal microscopy (Fig. 6 A-B). Under parallel conditions, BafA1 treatment rescued LD formation that was reduced by PFK158 in OVCAR8 cells (Fig. 6 C). Taken together, these results mechanistically support that PFK158-induced autophagy-mediated downregulation of PLA2G3 regulates PC in OC. By analyzing percent ciliated cells, we determined that BafA1 treatment downregulated acetylated α-tubulin in OVCAR5-sh35KD cells confirming the role of autophagy (Fig. 6D-E), which was also validated by western blot analysis, that showed a rescue of both LC3BII and p62 levels (Fig. 6 F). To validate the role of autophagy in regulating ciliogenesis, we determined acetylated α-tubulin levels in WT and in autophagy compromised Atg5^−/−^ MEFs, by IF. Atg5^−/−^ MEFs showed reduced percent ciliated cells compared to their WT counterpart (Fig. 6G), which was also corroborated by western analysis (Fig. 6 H, panel-2). Further, PFK158 treatment did not show a significant change in the expression of acetylated α-tubulin in Atg5^−/−^ MEFs (Fig. 6I, lower panel-1). In contrast there was a significant up-regulation in WT cells (Fig. 6I, top panel-1). Together, these results show PFK158-induced autophagy that leads to PLA2G3 degradation regulates ciliary maintenance.

### PFK158-mediates autophagic degradation of PLA2G3 and reduces viability in patient-derived ascites

**Fig. 7:**
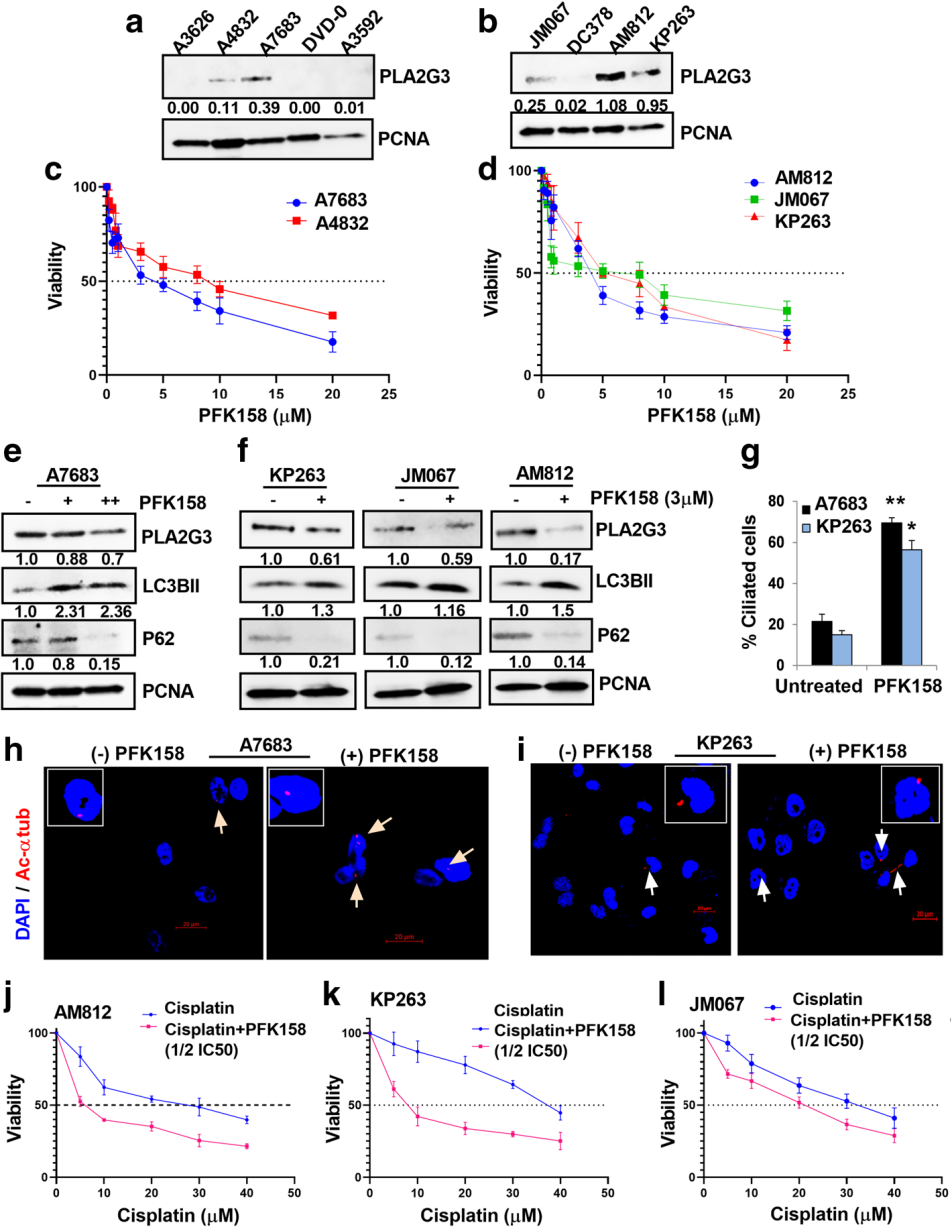
PFK158 treatment reduces cell viability in human patient-derived ascitic cells through autophagic degradation of PLA2G3. (A-B) Expression analysis of PLA2G3 levels in 9 patient-derived ascetic culture models. Densitometric analysis using Image J software was calculated, normalized to PCNA, and the fold change was provided beneath the panel. (C-D) Percent cell viability was assessed by MTT assay with an increasing concentration of PFK158 (0–20µM) in 5 ascitic cell models that express PLA2G3. IC50 value was calculated (A4832:IC50: 4µM, A7683:IC50: 9µM, JM076:IC50: 6.9µM, AM812:IC50: 4.1µM and KP263:IC50: 5.3µM). (E) Immunoblot analysis of A7683 cells upon treatment with 3 and 5µM PFK158 and (F) the mentioned other 3 ascites samples with 3µM PFK158 against PLA2G3, p62/SQSTM1and LC3BII. PCNA was probed for endogenous control. Image J software used for densitometric calculation, normalized to PCNA, and fold change was provided beneath the panel. (G) The percent ciliated cells in PFK158 treated A7683 and KP263 ascites cultures were quantified in 100 cells per field from the IF study and represented as mean ± SD (*p < 0.05, **p < 0.01 vs. control). (H-I) IF analysis of fluorescently tagged-acetylated α tubulin (red) to score the percent ciliated cells in A7683 and KP263 ascitic cells upon treatment with 3µM PFK158 for 24 h. DAPI was used to probe the nuclei. Images were captured using confocal microscopy. Scale Bar: 20 μm. (J-L) Percent cell viability was assessed by MTT assay with an increasing concentration of cisplatin (0–40µM) alone and combined with 1/2 IC50 concentration of PFK158 in the mentioned ascetic cell models and the shift in IC50 of cisplatin treatment was analyzed

To understand the clinical relevance, we determined PLA2G3 expression in 9 patient-derived ascites cells [27, 28]. Immunoblot analysis with human epithelial specific antigen marker (EpCAM) and fibroblast activated protein marker (FAP) showed that the ascitic cells are predominantly epithelial in nature (Fig. S5A) and 5 out of 9 samples expressed PLA2G3 (Fig. 7 A-B). A reduction in percent viability was observed in PLA2G3-expressing ascitic cells following PFK158 (0–20µM) treatment at 24 h with IC50 values ranging between 4.0–9.0µM (Fig. 7 C-D). Immunoblot analysis showed PFK158-induced autophagy, as determined by an increase in LC3BII, decreased p62 levels, and downregulation of PLA2G3 respectively (Fig. 7E-F). Further, IF analysis showed significant increase in percent ciliated cells upon PFK158-treatment in the A7683, KP263 and A4832 ascitic cells model compared to untreated control (Fig. 7G-I, S5B). When cisplatin was combined with 1/2IC50 of PFK158, a substantial reduction in IC50 ranging from 29µM to 7µM (AM812), 37.5µM to 8.5µM (KP263) and 33µM to 21µM (JM067; Fig. 7 J-L) was observed, suggestive of the ability of PFK158 to sensitize the cells to chemotherapy. To our interest we found that the AM812 and KP263 ascitic cells that expressed high levels of PLA2G3 showed increased sensitivity (lower IC50) to PFK158/Cisplatin combination treatment compared to the low expressing JM067 cells (Fig. 7 J-L). Similar observation was also obtained in both the OVCAR8 and OVCAR5 OC cells which showed sensitization (lower IC50) to the combination treatment (IC50 13.5µM to 8µM for OVCAR8 and 21µM to 10.5µM for OVCAR5; Fig. S5C-D). Together PFK158-treatment sensitizes the patient-derived OC ascites to chemotherapy at least in part through the degradation of PLA2G3.

## Discussion

Although aberrant expression of several human sPLA2s is reported in the pathogenesis of different cancers [13], the role of sPLA2s is controversial, since it can function either as a positive or negative regulator of tumorigenesis depending on the isoform, tissue/cancer types [15]. Current evidence suggests that high expression of PLA2G3 significantly correlates with metastasis and poor prognosis [36]. Therefore, a better understanding on the role and regulation of PLA2G3 in OC can open new therapeutic opportunities for targeting these enzymes.

Once secreted the sPLA2s can function either in an autocrine or paracrine manner, or as enzymes acting on extracellular/cellular phospholipid substrates to change the nature of FAs and lysophospholipids in tumor microenvironment [15, 37]. Current studies highlight the role of various sPLA2s in modulation of lipid metabolism, which add-ons to their functional complexities [38]. Recent development is focused on therapeutic peptides and small molecule inhibitors to inhibit the sPLA2 activity in different cancers [39]. Their potential as cancer biomarkers is well recognized [14], and due to their secretion into tumor microenvironment, can lead to the development of new target opportunities.

**Fig. 8:**
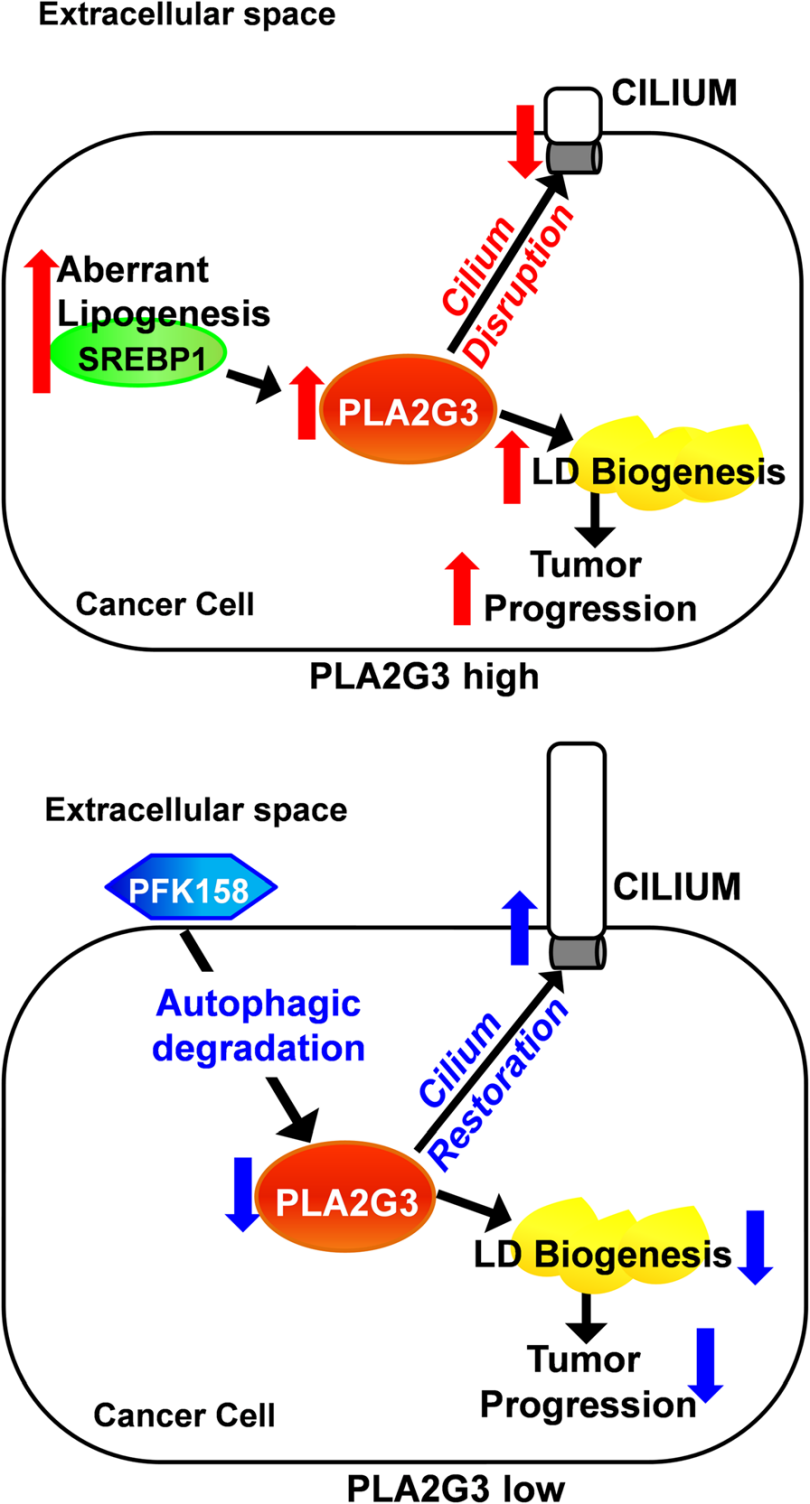
Mechanistic model showing the regulation of ovarian cancer progression by PLA2G3. Cancer cells show high lipogenic activity that promotes tumor progression. High levels of PLA2G3 are associated with reduced primary ciliogenesis and increased LD biogenesis in the cancer cells and thus promote the metastatic phenotype. Both PFK158 induced autophagy-mediated targeted degradation as well as the specific genetic knockdown of PLA2G3 restored primary ciliogenesis, sensitized cells to platinum drug treatment and attenuated the associated metastatic phenomenon

Increased *de novo* lipogenesis in transformed cells acts as an additional energy source for a high proliferative rate [38, 40, 41] and is associated with chemoresistance [10]. Reports suggest that LD-mediated resistance to chemotherapy is multifactorial and associated with poor prognosis [42, 43] and support the concept that targeting LDs alone or in combination with standard chemotherapy may lead to new clinical outcomes in cancers with lipogenic phenotype. We found a significant attenuation in LD biogenesis in PLA2G3-deficient OC cells. Since PC is associated with altered lipogenesis [26], and based on the role of PLA2G3 in ciliogenesis, we further explored whether PLA2G3 downregulation can restore PC in OC and can sensitize them to chemotherapy. PC is the regulatory signaling hub [44] and its loss in pre-invasive stages act as an early-oncogenic event in namely pancreatic adenocarcinoma [25]. Deng et al., reported the role of PC in sensitizing cells to transformation through mevalonate pathway activation indicating the regulation of metabolic plasticity in cancer and ciliogenesis [45]. In support, Gijs et al., reported that aberrant activation of SREBP1c suppresses cilia formation [26]. To this end, our present study suggests that PLA2G3 downregulation results in the PC restoration and sensitizes OC cells to platinum-drugs (Fig. 8). Additionally, we found that overexpression of SREBP1c, a direct transcriptional regulator of PLA2G3 [26], result in decrease in the percent ciliation which was further rescued when PLA2G3 was knocked down in the SREBP1c overexpressed cells. The outcome from our *in vivo* analysis of OVCAR5 xenografts showing substantial inhibition of tumor growth/metastasis was accompanied by an increase in acetylated α-tubulin in KO-derived xenograft compared to control and suggests the role of PLA2G3 towards OC metastasis partially through regulation of ciliogenesis.

Our findings provide novel insights into the abrogation of PLA2G3 towards sensitizing the OC cells to chemotherapy in part by downregulating LD biogenesis and restoring ciliogenesis. Our data add to current understanding on controversial interplay between PC and autophagy in other cancers. Tang et al., showed that autophagy promoted ciliogenesis by inducing degradation of a ciliogenic protein, oral-facial-digital syndrome-1(OFD1) [18]. This together with other studies suggests that autophagy positively regulates ciliogenesis [46]. In contrast, Pampliega et al., proposed that inhibition of autophagy induces PC and cilia-associated signaling [47]. Hence, the complex interplay between autophagy and PC varies depending on cancer cell types. Recently HDAC6 inhibitors were reported to restore PC and inhibit cholangiocarcinoma [48]. Our study in OC suggests that autophagy positively regulates ciliogenesis as PFK158-induced autophagic activity results in PC restoration. To validate autophagy-mediated regulation, we found that PFK158-treatment did not show a significant change in acetylated α-tubulin levels in Atg5-/- cells compared to its significant up-regulation in WT cells. More specifically, we show for the first time that targeting PLA2G3 by PFK158-induced autophagy plays a role in restoration of PC in OC cells and reduces tumor progression.

Importantly, PFK158 treatment of patient-derived ascites cells also reveals that PLA2G3 is degraded in an autophagy-dependent manner to sensitize cells to cisplatin and reduce cell viability, thus providing crucial *in vivo* support. Of relevance, our data showed that PFK158-induced acetylated α-tubulin levels are significantly higher in ascitic cells. We analyzed PLA2G3 expression between recurrent ovarian tumors (secondary debulking) and their autologous primary tumors (primary debulking) from 19 patients with triplicate cores on a tissue microarray (TMA) by IHC, and did not find statistically significant differences between the groups (data not shown). One limitation is small number of patient tumors analyzed which limits statistical power. Given our data that PLA2G3-KD inhibits metastasis *in vivo*, analyzing primary ovarian tumors vs. their autologous metastatic tumors (bowel/omental Mets) in patients, may be more informative to determine the role of PLA2G3 in the OC prognosis.

Taken together our findings become significant as many recent studies aim at identifying drugs that can be repurposed and used to restore ciliogenesis in cancer cells [49]. Thus, elucidation of pathways and molecular inhibitors towards ciliogenesis will be of interest to forge forward to be tested in a novel clinical setting.

## Conclusions

Our study in ovarian cancer provides significant novel insights into the down regulation of PLA2G3 towards sensitizing the cancer cells to chemotherapy (Fig. 8). Since ciliation is found to be regulated in an autophagy dependent process, this finding also paves the path for future analysis of the small molecule autophagy modulators that can have a promising impact on inhibiting OC progression as well.

## Supplementary Information

**Additional file 1.**

## Data Availability

Not applicable.

## Abbreviations

ATG: Autophagy-related
BafA1: Bafilomycin A1
LAMP2A: Lysosomal associated membrane protein type 2 A
LC3B: Microtubule associated protein 1 light chain 3 beta
shRNA: Short hairpin RNA
siRNA: Small interfering RNA
SQSTM1: Sequestosome 1
Ac-αtub: Acetylated α tubulin
PCNA: Proliferating Cell Nuclear Antigen
PC: Primary cilia
OC: Ovarian cancer
PLA2G3: Group III phospholipase A2 (group III sPLA2)
LDs: Lipid droplets
KO: Knock-out
KD: Knock-down
MDR: Multi drug resistant
IC50: Half maximal inhibitory concentration
SREBP1c: Sterol regulatory element-binding protein 1c
