# Supplementary material for: Group III phospholipase A2 downregulation attenuated survival and metastasis in ovarian cancer and promotes chemo-sensitization
Source: J Exp Clin Cancer Res. 2021 Jun 3;40:182. doi: 10.1186/s13046-021-01985-9 (PMC8173968; doi:10.1186/s13046-021-01985-9)
Supplement: Supplementary file 1 — Additional file 1. [file 13046_2021_1985_MOESM1_ESM.pdf]

Group III phospholipase A2 downregulation attenuated survival and metastasis in ovarian cancer and promotes chemo-sensitization

Upasana Ray[1], Debarshi Roy[1,2], Ling Jin[1], Prabhu Thirusangu[1], Julie Staub[1], Yinan Xiao[1], Eleftheria Kalogera[3], Andrea E. Wahner Hendrickson[4], Grace Cullen[4], Krista Goergen[5], Ann L. Oberg[5] and Viji Shridhar[1]*

[1]Department of Experimental Pathology and Medicine, Mayo Clinic, Rochester, MN, USA

[2]Alcorn State University, Lorman, MS, USA

[3]Division of Gynecologic Surgery, Mayo Clinic, Rochester, MN, USA

[4]Department of Internal Medicine, Division of Medical Oncology, Mayo Clinic, Rochester, MN, USA

[5]Department of Health Sciences Research, Division of Biomedical Statistics and Informatics, Mayo Clinic, Rochester, MN, USA

*Correspondence and requests for materials should be addressed to

Dr. Viji Shridhar
Department of Experimental Pathology and Laboratory Medicine,
Mayo Clinic College of Medicine,
Rochester, MN 55905, USA
Email: shridhar.vijayalakshmi@mayo.edu

Table S1:

Antibodies	Company	Catalog No.	Ab Dilutions
1. Cleaved PARP1	Cell Signaling Technology, Danvers, MA	cst5625	WB- 1:2000
2. LC3B	Cell Signaling Technology, Danvers, MA	cst3868	WB- 1:2000
3. P62	Santa Cruz Biotechnology, Texas, U.S.A	sc28359	WB- 1:1000
4. PCNA	Santa Cruz Biotechnology, Texas, U.S.A	sc9857	WB- 1:1000
5. Acetylated α tubulin	Santa Cruz Biotechnology, Texas, U.S.A	sc-23950	WB- 1:1000
6. PLA2G3	GeneTex, CA, U.S.A	GTX110780	WB- 1:1000<br>IF- 1:100
7. Cleaved caspase 3	Cell Signaling Technology, Danvers, MA	cst9664	WB- 1:2000
8. IFT88	Santa Cruz Biotechnology, Texas, U.S.A	sc-376680	WB- 1:1000
9. Ki67	Cell signaling Technology, Danvers, MA	cst9027	WB- 1:2000
10. GAPDH	Santa Cruz Biotechnology, Texas, U.S.A	sc-47724	WB- 1:1000
11. ATG5	Cell Signaling Technology, Danvers, MA	cst12994	WB- 1:2000
12. Acetylated α Tubulin Antibody (6-11B-1) Alexa Fluor® 594	Santa Cruz Biotechnology, Texas, U.S.A	sc-23950 AF594	IF- 1:100
13. Human epithelial specific antigen	Chemicon International, Temecula, CA, U.S.A	CBL251	WB- 1:1000
14. Fibroblast activated protein	R&D Systems, Inc., MN, U.S.A	AF3715	WB- 1:1000
15. SREBP1	Santa Cruz Biotechnology, Texas, U.S.A	sc-13551	WB- 1:1000

Reagents	Company	Catalog No.
1. IFT88 siRNA (h)	Santa Cruz Biotechnology, Texas, U.S.A	sc-75329
2. PLA2G3 siRNA (h)	Santa Cruz Biotechnology, Texas, U.S.A	sc-75201
3. Bodipy (493/503)	Sigma	790389
4. Baflomycin A1	Sigma	B1793
5. Cyto-ID® Autophagy Detection Kit	Enzo Life Sciences	ENZ-51031
6. 3-(4,5-dimethylthiazol-2-yl)-2,5-diphenyltetrazolium bromide (MTT)	ThermoFisher Scientific	M6494
7. Antifade mounting medium with DAPI	Vectashield, Burlingame, CA USA	H-1200-10
8. Carboplatin	TEVA UK limited	#55770169
9. Cisplatin	Calbiochem	#232120
10. Fetal Bovine Serum (FBS)	Biowest	#S181A
11. 100µg/ml streptomycin and 100U/ml penicillin	Thermo Fisher Scientific	15070063
12. MCDB-105	Sigma-Aldrich	M6395
13. Medium-199	Sigma-Aldrich	M4530
14. DMEM/F12,<br>15. DMEM (4.5 g/l glucose),<br>16. RPMI-1640,<br>17. MEM-α	Thermo Fisher Scientific	#11330032,<br>#11965118,<br>#11875093,<br>#12571063

18. Ultroser™ G serum substitute	Pall Corporation	15950-017
19. pcDNA3.1-2xFLAG-SREBP-1c	Addgene	#26802

Table S2:

Cell lines	Media	Supplements
OVCAR5	RPMI-1640	10% FBS and 1% Pen/Strep
OVCAR8	RPMI-1640	10% FBS and 1% Pen/Strep
PEO1	RPMI-1640	10% FBS and 1% Pen/Strep
OV202	RPMI-1640	10% FBS and 1% Pen/Strep
OVCAR7	DMEM (4.5 g/l glucose)	10% FBS, 1% Pen/Strep and insulin (0.25U/ml)
HeyA8MDR	DMEM (4.5 g/l glucose)	10% FBS and 1% Pen/Strep
MEF	MEM-α	10% FBS and 1% Pen/Strep
NOF151hTERT	MCDB-105: Medium-199 (1:1)	10% FBS and 1% Pen/Strep
FTs 240, 194, 190, 257	DMEM/F12 (1:1)	2% Ultroser G and 1% Pen/Strep
Patient-derived ascites	DMEM/F12 (1:1)	15%FBS and 1% Pen/Strep

Fig. S1. Downregulation of PLA2G3 attenuates OC migration. (A) Percent PLA2G3 gene expression altered in serous ovarian cancer as evaluated by TCGA analysis 2018. (B) Wound healing assay was performed to analyze the migration capability of OVCAR8 KO and SCG-control cells for 24hrs and in (C) OVCAR5 sh35 KD cells compared to NTC control transfected cells for 24 and 48hrs. (D) Confocal imaging shows Bodipy staining for LD biogenesis in OVACR5 sh33 and sh35 KD cells compared to NTC controls. DAPI was used to stain nucleus and Oleic acid treatment was used as a positive control (Panel 1).

Fig. S2. KD of PLA2G3 sensitizes HeyA8 MDR cells to CBP treatment. (A) Immunoblot shows efficient KD of PLA2G3 in the HeyA8 MDR cells. PCNA used as loading control. Percent cell viability as assessed by MTT assay in (B) HeyA8 MDR NTC control and (C) sh35 KD cells with increasing concentration of carboplatin treatment. IC50 values indicate PLA2G3 KD HeyA8MDR cells are more sensitive to CBP treatment. (D) Graphical representation of percent surviving cells from the above analysis (*$p<0.05$, **$p<0.01$).

Fig. S3. Abrogation of primary cilia promotes oncogenesis in OC cells. (A) Western blot analysis shows efficient siRNA mediated KD of IFT88 resulting in the downregulation of acetylated α-tubulin in OVCAR5 cells. PCNA was used as a loading control. (B) Colony forming potential was assessed in the NTC and IFT88 KD OVCAR5cells. (C) The number of colonies was counted and plotted as mean ± standard deviation ($n = 3$, **$p<0.01$). (D) Wound healing assay was performed in the NTC and IFT88 KD OVCAR5cells and (E) the migration rate quantification at 0hr and 24hr was plotted (**$p<0.01$). (F) Expression analysis of SREBP1 by western blot in fallopian tube epithelial cell lines FTs 257, 190 and 194 and the OVCAR8 OC cells with PCNA as a loading control. Densitometric analysis showing fold change was calculated using Image J software, normalized, and provided beneath the panel. (G) Western blot analysis of PLA2G3 was

shown in fallopian tube epithelial cell lines FT 257 and 190. (H) Primary cilia were detected by IF using fluorescently tagged-acetylated α-tubulin (red) in the EV transfected control and SREBP1c overexpressed FT257 cells with nontargeting siRNA (siNTC) and the PLA2G3 targeting siRNA (siPLA2G3). Nuclei were stained with DAPI. Scale bar: 10µm.

Fig. S4. PFK158 treatment restores ciliogenesis. (A) Representative confocal imaging of fluorescently tagged-acetylated α-tubulin (red) in OVCAR5 cells upon PFK158 treatment. DAPI was used to stain nucleus. (B) Western blot analysis of acetylated α-tubulin levels upon treatment with BafA1 (50 and 100nM) and 3MA (5µM) with PCNA as loading control. Fold change was calculated using the Image J software, normalized to PCNA endogenous control and provided beneath the panel.

Fig. S5. PFK158 treatment reduces cell viability in patient-derived ascites cells. (A) Immunoblot analysis of expression of human epithelial specific marker EpCAM and fibroblast marker FAP for characterization of ascites samples. GAPDH is used as endogenous control. (B) Confocal imaging of fluorescently tagged-acetylated α-tubulin (red) was assessed in the A4832 ascitic cells upon treatment with PFK158. DAPI was used to stain nucleus. Quantitation of percent ciliated cells was represented. (C) Percent cell viability as assessed by MTT assay in OVCAR8 and (D) OVCAR5 cells with increasing concentration of cisplatin (0-40µM) alone and combined with 1/2 IC50 concentration of PFK158 in the mentioned cells and the shift in IC50 of cisplatin treatment was analyzed.

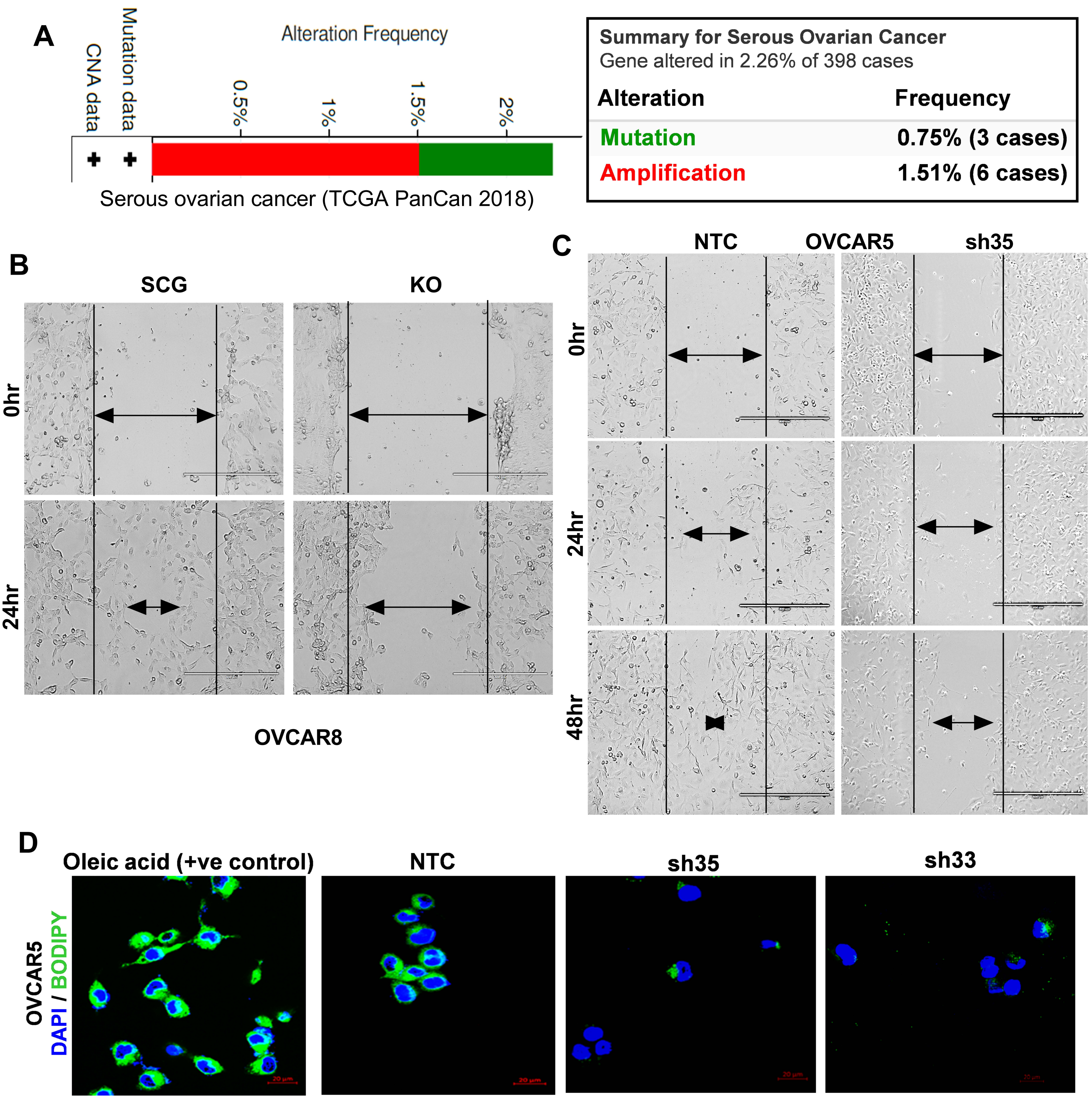
A
Alteration Frequency
CNA data
Mutation data
0.5%
1%
1.5%
2%
Serous ovarian cancer (TCGA PanCan 2018)
Summary for Serous Ovarian Cancer
Gene altered in 2.26% of 398 cases
Alteration
Frequency
Mutation
0.75% (3 cases)
Amplification
1.51% (6 cases)
B
SCG
KO
0hr
24hr
OVCAR8
C
NTC
OVCAR5
sh35
0hr
24hr
48hr
D
Oleic acid (+ve control)
NTC
sh35
sh33
OVCAR5
DAPI / BODIPY

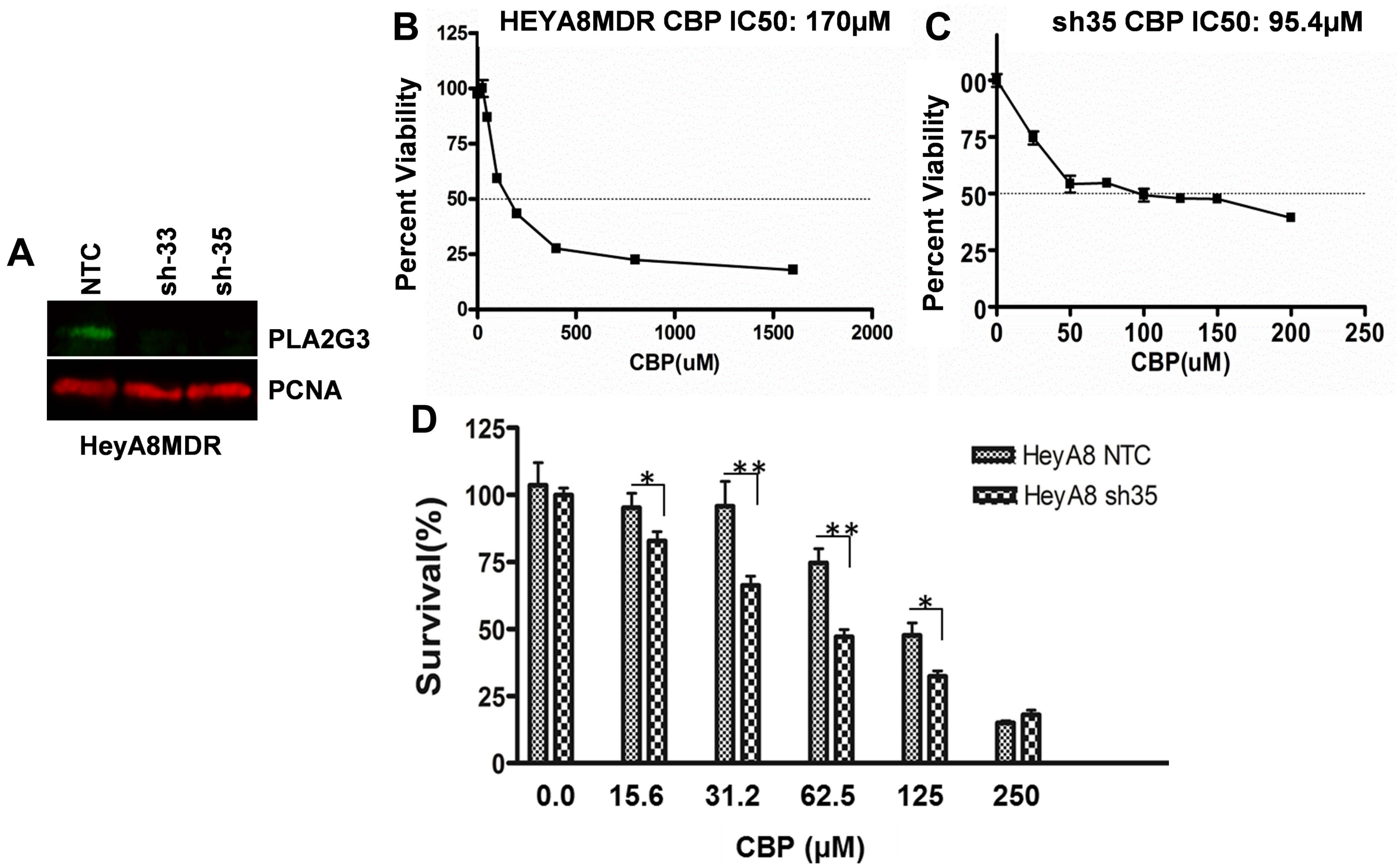

A
NTC
sh-33
sh-35
PLA2G3
PCNA
HeyA8MDR
B
HEYA8MDR CBP IC50: 170μM
Percent Viability
CBP(uM)
C
sh35 CBP IC50: 95.4μM
Percent Viability
CBP(uM)
D
HeyA8 NTC
HeyA8 sh35
Survival(%)
0.0
15.6
31.2
62.5
125
250
CBP (μM)

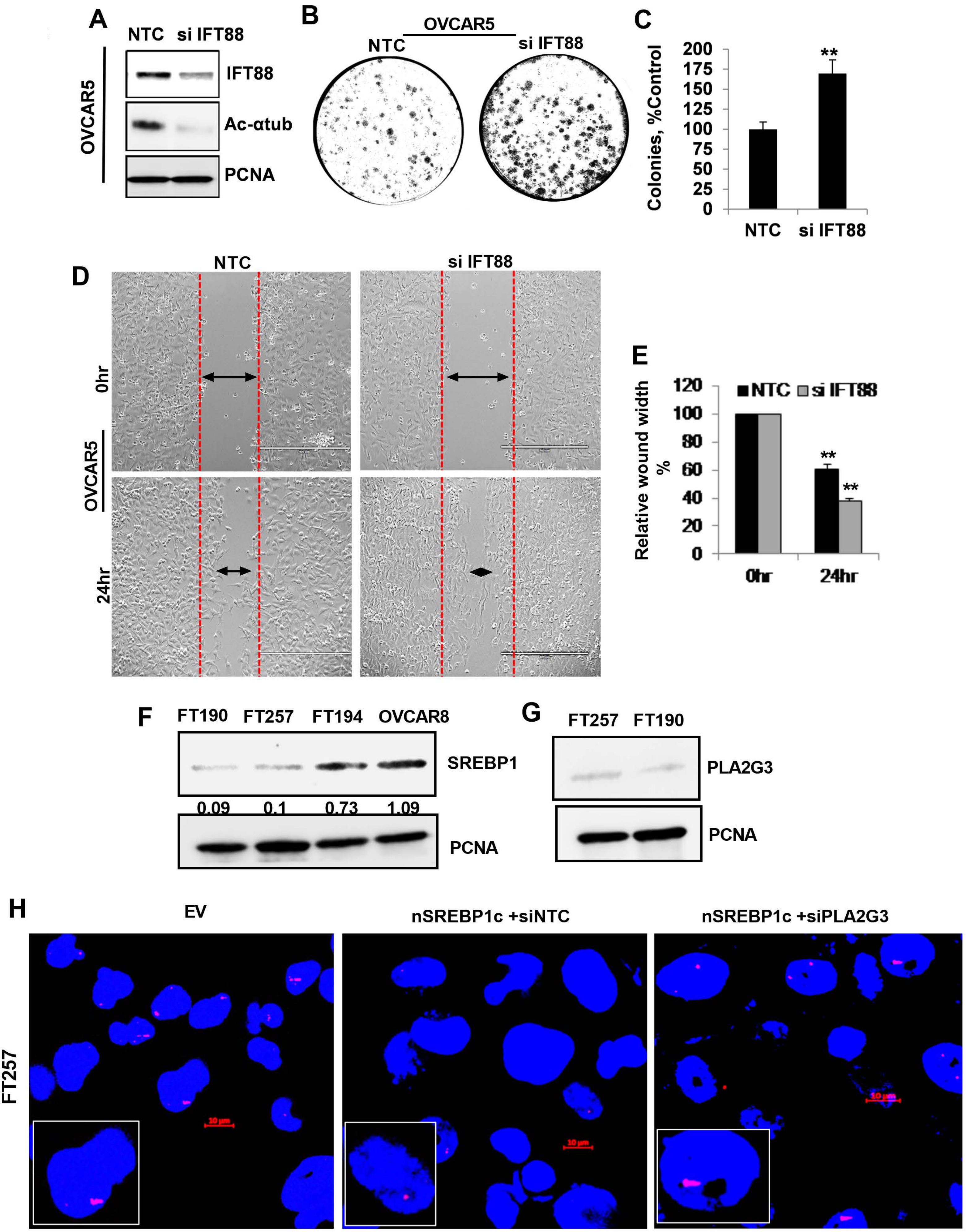
A
NTC
si IFT88
OVCAR5
IFT88
Ac-αtub
PCNA
B
OVCAR5
NTC
si IFT88
C
Colonies, %Control
200
175
150
125
100
75
50
25
0
**
NTC
si IFT88
D
NTC
si IFT88
0hr
OVCAR5
24hr
E
Relative wound width %
120
100
80
60
40
20
0
NTC
si IFT88
**
**
0hr
24hr
F
FT190
FT257
FT194
OVCAR8
SREBP1
0.09
0.1
0.73
1.09
PCNA
G
FT257
FT190
PLA2G3
PCNA
H
EV
nSREBP1c +siNTC
nSREBP1c +siPLA2G3
FT257
10 μm

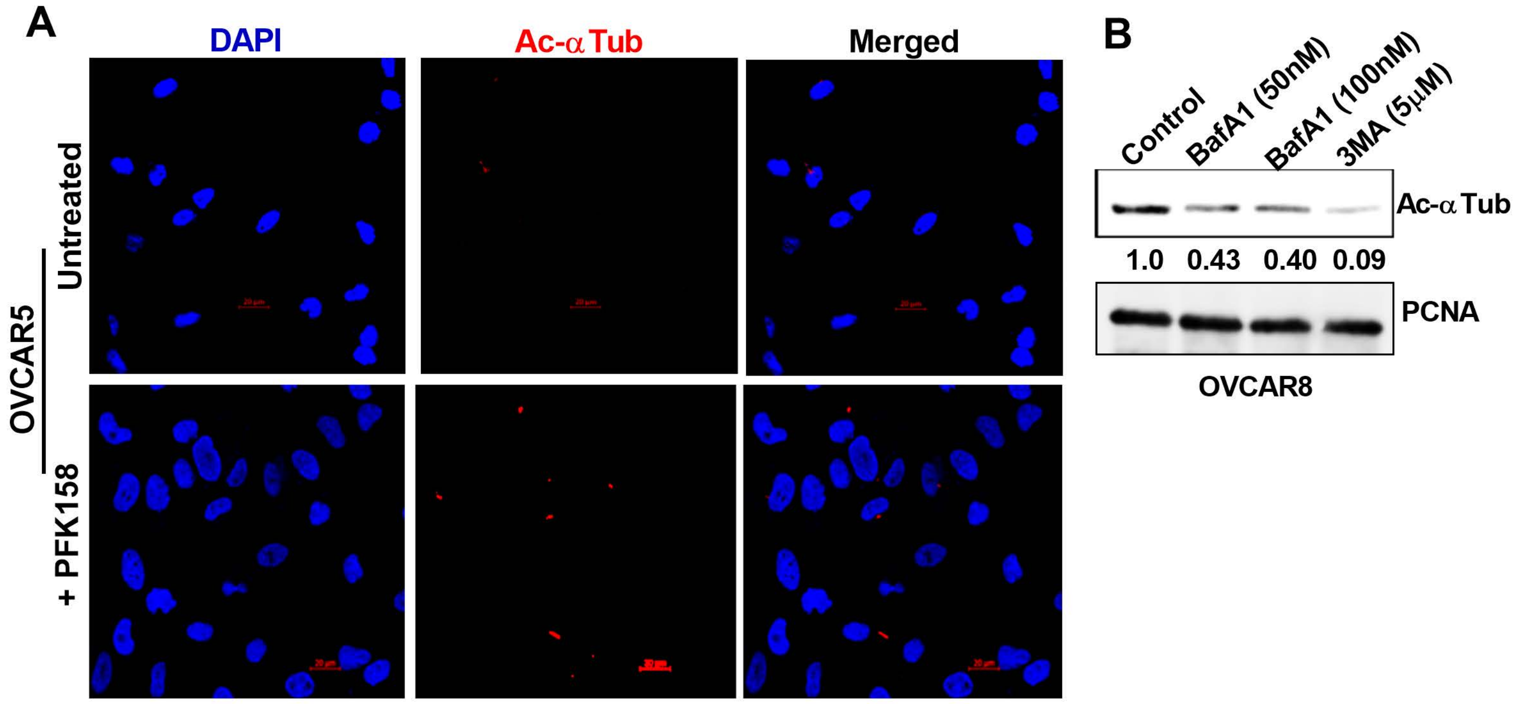
A
DAPI
Ac-α Tub
Merged
OVCAR5
Untreated
+ PFK158
B
Control
BafA1 (50nM)
BafA1 (100nM)
3MA (5μM)
Ac-α Tub
1.0
0.43
0.40
0.09
PCNA
OVCAR8

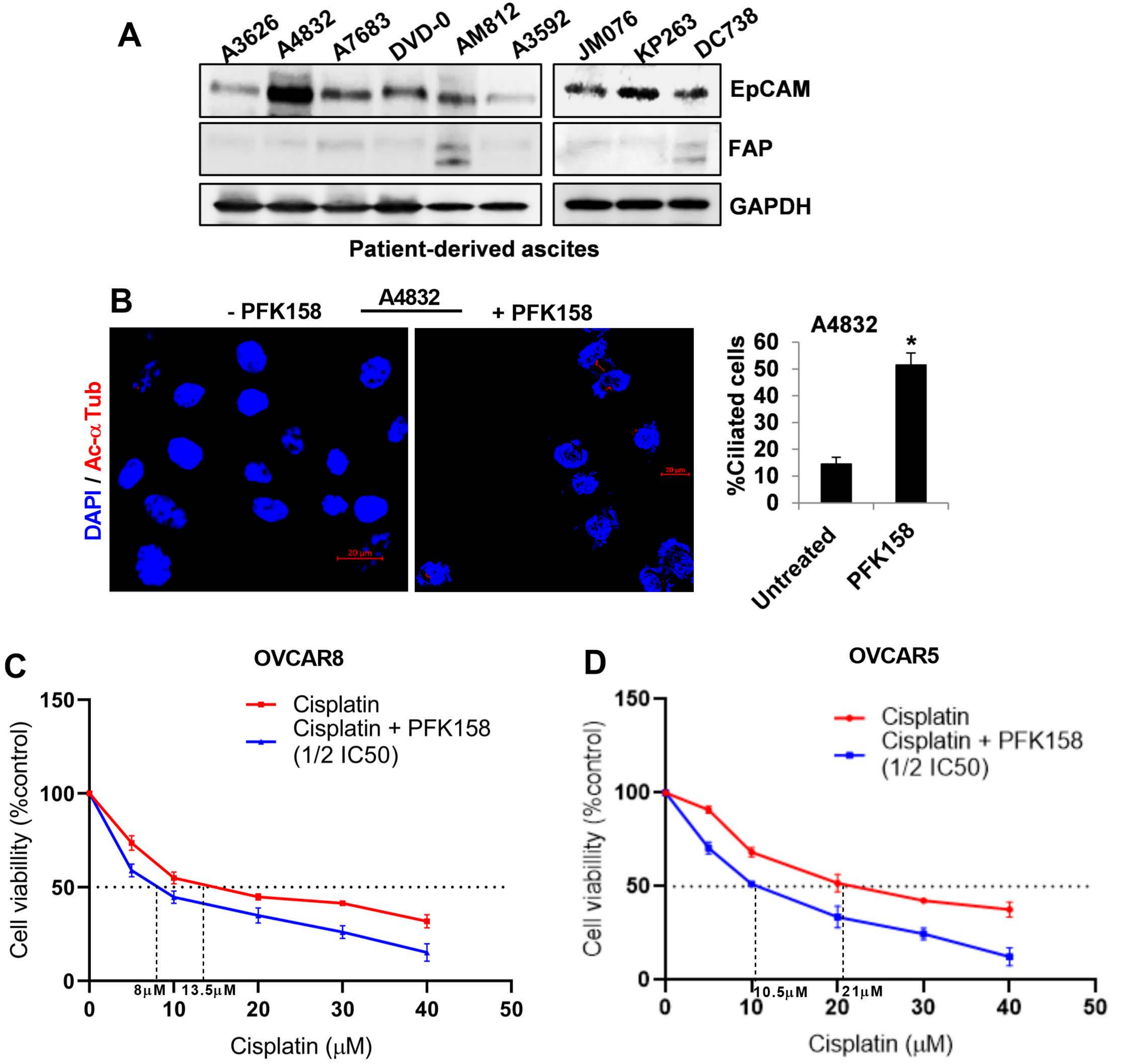

A
A3626
A4832
A7683
DVD-0
AM812
A3592
JM076
KP263
DC738
EpCAM
FAP
GAPDH
Patient-derived ascites
B
- PFK158
A4832
+ PFK158
DAPI / Ac-α Tub
20 μm
A4832
%Ciliated cells
60
50
40
30
20
10
0
*
Untreated
PFK158
C
OVCAR8
Cisplatin
Cisplatin + PFK158 (1/2 IC50)
Cell viability (%control)
150
100
50
0
8μM
13.5μM
0
10
20
30
40
50
Cisplatin (μM)
D
OVCAR5
Cisplatin
Cisplatin + PFK158 (1/2 IC50)
Cell viability (%control)
150
100
50
0
10.5μM
21μM
0
10
20
30
40
50
Cisplatin (μM)
